# Positional change in mandibular condyle in facial asymmetric patients after orthognathic surgery: cone-beam computed tomography study

**DOI:** 10.1186/s40902-018-0152-6

**Published:** 2018-06-25

**Authors:** Byung-Joon Choi, Byung-Soo Kim, Ji-Min Lim, Junho Jung, Jung-Woo Lee, Joo-Young Ohe

**Affiliations:** Department of Oral and Maxillofacial Surgery, School of Dentistry, Kyung Hee University, Seoul, Republic of Korea

**Keywords:** Asymmetry, Condyle position, CBCT

## Abstract

**Background:**

We evaluated change in the mandibular condyle after orthognathic surgery using cone-beam computed tomography (CBCT) in patients with facial asymmetry.

**Methods:**

Thirty patients with skeletal class III malocclusion and mandibular prognathism or facial asymmetry were classified into two groups according to the amount of menton deviation (MD) from the facial midline on anteroposterior (AP) cephalogram: group A (asymmetry, MD ≥ 4 mm; *n* = 15) and group B (symmetry, MD < 4 mm; *n* = 15). Position and angle of condylar heads on the axial, sagittal, and coronal views were measured within 1 month preoperatively (T0) and postoperatively (T1) and 6 months (T2) postoperatively.

**Results:**

On axial view, both groups showed inward rotation of condylar heads at T1, but at T2, the change was gradually removed and the condylar head returned to its original position. At T1, both groups showed no AP condylar head changes on sagittal view, although downward movement of the condylar heads occurred. Then, at T2, the condylar heads tended to return to their original position. The change in distance between the two condylar heads showed that they had moved outward in both groups, causing an increase in the width between the two heads postoperatively. Analysis of all three-dimensional changes of the condylar head positions demonstrated statistically significant changes in the three different CBCT views in group B and no statistically significant changes in group A.

**Conclusions:**

There was no significant difference between the two groups in condylar head position. Because sagittal split ramus osteotomy can be performed without significant change in symmetrical and asymmetrical cases, it can be regarded as an effective method to stabilize the condylar head position in patients with skeletal class III malocclusion and mandibular prognathism or facial asymmetry.

## Background

Orthognathic surgery is a commonly conducted surgical procedure to improve nonesthetic facial appearance and accomplish more stable and functional balance on the masticatory system with optimal occlusion. Bilateral sagittal split ramus osteotomy (BSSRO) divides the mandible into proximal and distal segments to allow movement of these segments such that the relocation of pieces on more optimal positions alleviates or removes the functional and esthetic issues. In that sense, if the current condylar head positions are proper and stable, maintaining the original positions of the condylar heads is crucial to re-establish new occlusion by surgery. However, some unexpected changes in condylar head position and the distance between the medial poles of condylar heads on both sides can occur during open relocation of the proximal and distal segments and rigid fixation between the two separated bone parts during intermaxillary fixation [1].

Good occlusal relation and clinically normal locations of condylar heads are significant factors to prevent relapse after BSSRO. The two factors seem independent, but abnormal occlusion can be corrected postoperatively by correctional treatments, including postoperative orthodontic measures when normal condyle head location is assured. On the other hand, modification treatment to set a better occlusal relationship postoperatively is impossible because additional changes of the condyle will always ruin the safety of the new occlusion set by correctional treatment postoperatively if the condylar head locations are unstable or are not optimal. Postoperative condyle head location can vary depending on various factors, such as skill level of the surgeon, amount and direction of displacement of the distal from the proximal segments on the surgical plan, anatomic shape of the proximal segment, and fixation method [2, 3].

Asymmetric movement of the distal segment occurs in patients with facial asymmetry, and outward displacement of the condylar head can occur. Although Lee and Park et al. [2] reported that setback amount of the mandible does not have a statistically significant influence on movement of the condyle head, Baek et al. [4] reported that the condylar head is displaced backward and rotated inward after an asymmetric setback movement of the distal segment of the mandible, and the amount of backward displacement of the condylar heads on both sides in the sagittal plane was different. The condylar head on the larger setback side was more posteriorly positioned than that on the lesser setback side.

It is difficult to analyze condyle head locations just by plain radiographs, such as transcranial or panorama views of the temporomandibular joints, because anatomic structures can overlap with surrounding structures to cause illusions or artificial shadows. The images are two-dimensional, and it is almost impossible to reproducibly locate the patient’s head to get standardized results. On the other hand, computed tomography (CT) can avoid overlapping of anatomic structures and allows observation of more detailed structures so that surgeons can evaluate the condylar head location more accurately and reproducibly. Research methods on temporomandibular joints using three-dimensional (3D) CT have been reported to have 0.5 mm less average variability compared with methods using plain radiographs [5]. Moreover, Katsumata et al. reported that values measured by 3D CT were almost equivalent to actually measured values and that the method is very useful for precise maxillofacial measurement [6].

We used cone-beam CT (CBCT) to analyze anteroposterior (AP), superoinferior, and mediolateral locations and angles of condylar heads defined by our group within 1 month preoperatively (T0) and postoperatively (T1) and 6 months (T2) postoperatively to evaluate changes in condylar heads after BSSRO.

## Methods

### Subjects

We studied 30 patients (19 males, 11 females; age range, 18–25 years; average age, 22 years) who underwent orthognathic surgery at the Oromaxillofacial Surgery Department of Kyung Hee University Dental Hospital between 2010 and 2012 because of prognathism or facial asymmetry. Methods of surgery were BSSRO only in 7 patients and BSSRO plus Le Fort I osteotomy in 23. In all patients, the direction of movement of the distal segment on BSSRO was backward, and the fixation methods of bone segments on Le Fort I osteotomy and BSSRO were rigid fixation methods by rigid mini-plates and monocortical screws.

The subjects were classified into two groups depending on the degree of menton deviation (MD) from the facial midline on AP cephalogram. Patients with ≥ 4 mm MD were assigned to group A (*n* = 15) and those with < 4 mm MD were assigned to group B (*n* = 15) [7, 8].

### Data acquisition

The panoramic mode of CBCT was used for each temporomandibular joint area in all patients at T0, T1, and T2. The Alphard-Vega 3030 Dental CT system (Asahi Roentgen Ind. Co., Ltd., Kyoto, Japan) was used for CBCT. The head was fixed with a head fixation device so that the Frankfort horizontal plane of the patient would be parallel to the ground, and images were obtained in the panoramic mode. Tube voltage was 80 kVp, tube current was approximately 5–10 mA, and time of exposure was 17 s. Cross-section thickness of the images was 0.3 mm, and images of equivalent parts were taken at every session.

The raw data of CBCT obtained through this process were evaluated using the InVivoDental^®^ (Anatomage Inc., San Jose, CA, USA) program. Distances among the main anatomic structures and shapes of the parts were measured from the axial, sagittal, and coronal planes. The measurement method used by Ueki et al. [9–11] was referred to for accurate measurement.

Each procedure was performed with approval from the Kyung Hee University Life Ethics Institutional Review Board (assignment number, KHD IRB 1311-2).

**Fig. 1:**
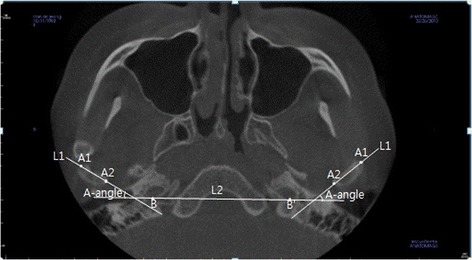
Reference points and measurement of axial view in CBCT. A1 outer pole of condylar head, A2 inner pole of condylar head, B,B′ hindmost point of the carotid canal, L1 line passing A1 and A2, L2 line passing B and B′, A-angle angle between L1 and L2

By selecting the slice where the maximum mediolateral width of a condylar head is seen from the axial view, the axial condylar head angle (A-angle; L1,L2) was measured by the intersection between the line (L1; A1–A2) connecting the outermost (A1) and the innermost (A2) side points and the line (L2; B–B′) connecting the hindmost points of both carotid canals (right side, B; left side, B′; Fig. 1).

**Fig. 2:**
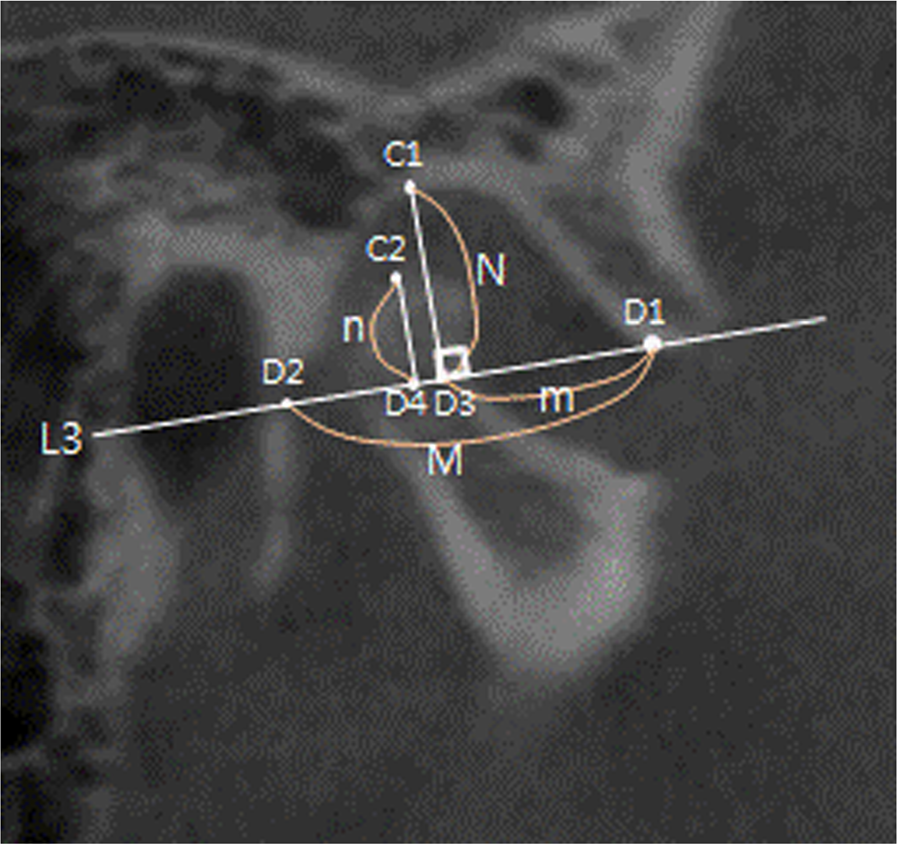
Reference points and measurement of sagittal view in CBCT. C1 highest point of glenoid fossa, C2 highest point of condylar head, D1 lowest point of articular eminence, D2 lowest point of temporal squamotympanic fissure, L3 line passing D1 and D2, D3 point meeting the perpendicular line from C1 on L3, D4 point meeting the perpendicular line from C2 to L3, *M* distance between D1 and D2, *m* distance between D1 and D3, *N* distance between C1 and D3, *n* distance between C2 and D4, S–N *n*/*N* (assessed by ratio of vertical location of mandibular condyle at glenoid fossa), S–M, *m*/*M* (assessed by ratio of horizontal location of mandibular condyle at glenoid fossa)

In the sagittal view, a line (L3; D1–D2) connecting the most inferior point (D1) of the articular eminence and the bottom point (D2) of the osseous entrance of the temporal squamotympanic fissure was drawn. Vertically, the distance (*N*; C1–D3) between the highest point (C1) of the glenoid fossa and the point (D3) that meets with the perpendicular line from C1 on L3 was measured, and the distance (*n*; C2–D4) between the highest point (C2) of the condylar head and the point that meets with the perpendicular line from C2 on L3 was measured to find the S–N value, calculated by *n*/*N*. Horizontally, the distance (*M*; D1,D2) between the two points D1–D2 and the distance (*m*; D1–D3) between the two points D1–D3 were measured, and then, the S–M value was calculated by *m*/*M* (Fig. 2).

**Fig. 3:**
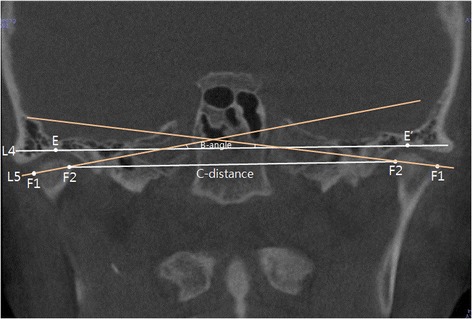
Reference points and measurement of coronal view in CBCT. E,E′ highest point with joint, F1 outer pole of condylar head, F2 inner pole of condylar head, L4 line passing E and E′, L5 line passing F1 and F2, B-angle angle between L4 and L5, C-distance distance between left F2 and right F2

The slice where the maximum mediolateral width of the condylar head was seen at the coronal view was selected to measure the coronal condylar head angle (B-angle) intersecting the line (L5; F1–F2) connecting the outermost (F1) and the innermost (F2) points of the condylar head and the line (L4; E–E′) connecting the highest points (E,E′) of both glenoid fossae. In addition, the distance between the coronal condylar heads (C-distance; right side, F2; left side, F2) was measured by connecting the innermost points of both condylar heads (Fig. 3).

### Statistical analysis

Mean values and standard deviations of A-angle, S–N, S–M, B-angle, and C-distance values were determined at T0, T1, and T2, and the changes between T0 and T1, T1 and T2, and T0 and T2 were compared. SPSS (v18.0) was used for statistical processes, and the paired *t* test was used to test significance at 95% significance level.

## Results

MD was measured on AP cephalogram, and the equivalent researcher conducted the measurements. Measured points were determined by the method proposed by Sassouni [12] and Ricketts [13]. Mean MD was 6.9 ± 4.27 mm in group A and 1.5 ± 0.87 mm in group B.

### Change in condylar head location in the axial plane

A-angle increased from 20.63° ± 5.98° and 18.13° ± 8.09° at T0 in groups A and B, respectively, to 22.93° ± 7.16° and 20.33° ± 7.87°, respectively, at T1. Then, it decreased to 20.80° ± 6.72° and 18.23° ± 7.71° at T2, respectively. Degree of change between T1 and T0, T2 and T1, and T2 and T0 was not statistically significantly different in either group (*P* < 0.05; Tables 1 and 2).

**Table 1:** Average position of the condyle on the axial view

	T0		T1		T2	
	Mean	S.D.	Mean	S.D.	Mean	S.D.
A-angle(group A)	20.63	5.98	22.93	7.16	20.80	6.72
A-angle(group B)	18.13	8.09	20.33	7.87	18.23	7.71
Total	19.38	7.11	21.63	7.51	19.52	7.23

**Table 2:** Positional changes of the condyle on the axial view

	T1–T0			T2–T1			T2–T0		
	Mean	S.D.	*P* value	Mean	S.D.	*P* value	Mean	S.D.	*P* value
A-angle(group A)	2.30	6.54	0.195	− 2.13	6.70	0.239	0.17	4.76	0.894
A-angle(group B)	2.20	3.36	0.054	− 2.10	4.00	0.067	0.10	2.63	0.881
Total	2.25	5.33	0.160	− 2.12	5.60	0.121	0.14	3.78	0.846

*P < 0.05 statistically significant changes

### Change in condylar head location in the sagittal plane

S–M in group A was 0.54 ± 0.05 at T0 and T1 without change and increased slightly to 0.56 ± 0.04 at T2. The degree of change between T1 and T0, T2 and T1, and T2 and T0 showed no statistically significant difference. S–N decreased from 0.66 ± 0.09 at T1 to 0.58 ± 0.13 at T2 and increased again to 0.62 ± 0.15 at T3. The degree of change was significantly different between T1 and T0 but not between T2 and T1 and T2 and T0.

In group B, S–M decreased slightly from 0.56 ± 0.04 at T0 to 0.53 ± 0.04 at T1 and increased again to 0.54 ± 0.04 at T2. The differences in the degree of change between T1 and T0, T2 and T1, and T2 and T0 were not statistically significant. S–N decreased slightly from 0.66 ± 0.11 at T0 to 0.60 ± 0.12 at T1 and increased again to 0.67 ± 0.11 at T2. The degree of change was significantly different between T1 and T0 but not between T2 and T1 and T2 and T0 (*P* < 0.05; Tables 3 and 4).

**Table 3:** Average position of the condyle on the sagittal view

	T0		T1		T2	
	Mean	S.D.	Mean	S.D.	Mean	S.D.
S-M(group A)	0.54	0.05	0.54	0.05	0.56	0.04
S-M(group B)	0.56	0.04	0.53	0.04	0.54	0.04
Total	0.55	0.05	0.54	0.04	0.55	0.04
S-N(group A)	0.66	0.09	0.58	0.13	0.62	0.15
S-N(group B)	0.66	0.11	0.60	0.12	0.67	0.11
Total	0.66	0.10	0.59	0.13	0.64	0.13

**Table 4:** Positional changes of the condyle on the sagittal view

	T1–T0			T2–T1			T2–T0		
	Mean	S.D.	*P* value	Mean	S.D.	*P* value	Mean	S.D.	*P* value
S-M(group A)	0.00	0.03	0.591	0.01	0.03	0.173	0.02	0.04	0.116
S-M(group B)	− 0.02	0.04	0.060	0.01	0.03	0.371	− 0.02	0.03	0.055
Total	− 0.01	0.04	0.208	0.01	0.03	0.098	0.00	0.04	0.946
S-N(group A)	− 0.08	0.06	0.000*	0.04	0.10	0.182	− 0.05	0.09	0.066
S-N(group B)	− 0.06	0.07	0.005*	0.07	0.60	0.083	0.01	0.03	0.218
Total	− 0.07	0.07	0.000*	0.05	0.46	0.056	− 0.02	0.07	0.184

*P < 0.05 statistically significant changes

### Change in condylar head location in the coronal plane

B-angle decreased slightly from 11.04° ± 3.83° and 10.62° ± 4.20° at T0 in groups A and B, respectively, to 10.10° ± 4.35° and 9.60° ± 4.51°, respectively, at T1 and increased again to 10.50° ± 6.02° and 10.39° ± 5.03°, respectively, at T2. In both groups, a significant difference was shown in the degree of change between T1 and T0 and T2 and T1 but not between T2 and T0 (*P* < 0.05; Tables 5 and 6).

**Table 5:** Average position of the condyle on the coronal view

	T0		T1		T2	
	Mean	S.D.	Mean	S.D.	Mean	S.D.
B-angle(group A)	11.04	3.83	10.10	4.35	10.50	6.02
B-angle(group B)	10.62	4.20	9.60	4.51	10.39	5.03
Total	10.83	3.95	9.85	4.36	10.45	5.45
C-distance(group A)	87.93	4.92	88.68	5.90	88.56	5.18
C-distance(group B)	86.05	4.19	87.78	4.67	86.23	5.20
Total	86.99	4.59	88.23	5.25	87.39	5.24

**Table 6:** Positional changes of the condyle on the frontal view

	T1–T0			T2–T1			T2–T0		
	Mean	S.D.	*P* value	Mean	S.D.	*P* value	Mean	S.D.	*P* value
B-angle(group A)	− 0.94	2.75	0.005*	0.4	3.77	0.000*	− 0.53	4.85	0.182
B-angle(group B)	− 1.02	3.11	0.003*	0.8	2.25	0.017*	− 0.22	2.87	0.881
Total	− 0.98	2.88	0.000*	0.6	3.06	0.000*	− 0.38	3.92	0.601
C-distance(group A)	0.75	2.64	0.287	− 0.13	3.37	0.887	0.63	3.13	0.451
C-distance(group B)	1.73	1.44	0.000*	− 1.55	2.14	0.014*	0.18	1.69	0.688
Total	1.24	2.15	0.004*	− 0.84	2.87	0.120	0.40	2.48	0.381

*P < 0.05 statistically significant changes

C-distance increased from 87.93 ± 4.92 and 86.05 ± 4.19 mm at T0 in groups A and B, respectively, to 88.68 ± 5.90 and 87.78 ± 4.67 mm, respectively, at T1 and decreased again to 88.56 ± 5.18 and 86.23 ± 5.20 mm, respectively, at T2. In group A, the degree of change between any of the three time points showed no statistically significant difference (*P* < 0.05), whereas in group B, there was a significant difference between T1 and T0 and T2 and T1 but not between T2 and T0 (*P* < 0.05; Tables 5 and 6).

## Discussion

Sagittal split ramus osteotomy is a representative operative technique used for functional and esthetic improvement in patients with prognathism and facial asymmetry. It has well-known advantages, such as easy relocation of the distal segment and a wide contact area between separated and relocated jawbone so that quick bone healing can occur. However, there are disadvantages, such as nerve damage, blood vessel damage, or condylar head displacement, so that the possibility of relapse postoperatively would be high. When conducting rigid fixation of the distal segment onto the proximal segment during sagittal split ramus osteotomy, inward rotation of the proximal segment occurs easily, and this leads to horizontal outward displacement of the condylar head [14].

Imamura reported that the change in the condylar head location could be originated from the surgical procedure itself [15]. Moreover, it is influenced by the posture of the patient, tension of masticatory muscles, type of osteotomy, fixation method, and method of locating the proximal segment, and a device that maintains condylar position was used for these reasons. However, maintaining accurate location of the condylar head is difficult, but it is recommended that the fixation should not be too firm. Hollender et al. [16] reported on 25 patients who underwent sagittal split ramus osteotomy because of prognathism, and position change in the condylar head, especially anterior and inferior movement, occurred.

Because of this change in condylar head location, functional change, disorder of the temporomandibular joint, occlusion disorder, and relapse due to segmental movement can occur, and the condylar head must be located similar to the preoperative position to prevent side effects and relapse [17, 18].

Hu et al. [19] conducted mandibular setback using sagittal split ramus osteotomy on 22 patients and reported that the condylar head was displaced backward and was rotated forward as seen on a temporomandibular joint radiograph. Kawamata et al. [20] used a jaw bone model manufactured using CT of patients with prognathism after sagittal split ramus osteotomy and evaluated the location of the condylar head preoperatively and postoperatively. They reported that the condylar head moved backward approximately 1–2 mm with an average increased distance between the condylar heads of 2 mm.

In this study, the 3D change in condylar head at T0, T1, and T2 in patients with skeleton class III malocclusion undergoing sagittal split ramus osteotomy using CBCT images was compared by classifying subjects into groups A and B (≥ 4 and < 4 MD, respectively). As a result of the research, the patterns of location and angle change in the condylar head in both groups were identical.

Regarding change in condylar head angle in the axial plane, Lee et al. [2] reported that an average of 4.00° condylar head rotation occurs in the axial plane and the proximal segment inwardly rotates postoperatively. Ueki et al. [21, 22] reported that inward or outward rotation of the condylar head occurs in the axial plane, and inward rotation of the condylar head is more common when firm fixation is conducted. Kim et al. [23] reported that condylar head angle in the axial plane increased by 2.23° on the right side and by 2.18° on the left side. Nishimura [14] stated that the outer point of the condylar head rotates anteromedially because of inner fixation of the proximal segment in patients with prognathism and that less change was shown in nonrigid than rigid fixation. In this study, both groups showed an A-angle increase (by 2.30° ± 6.54°, 2.20° ± 3.36° in each group), and this result was similar to previous results of other studies in which it is seen to be due to inward rotation of the condylar head as the screw is inserted during rigid fixation. The angle decreased at T2, and the condyle was considered to be returning to its original location. Regarding condylar head angle (A-angle) in the axial plane, the amounts of change between T1 and T0, T2 and T1, and T2 and T0 all showed no statistically significant difference, and patterns of change in the two groups were identical.

Regarding upper–lower and AP changes in the sagittal plane, Lee et al. [2] reported that the condylar head moved downward at an average of 0.36 mm and forward at 0.3 mm. S–M, which shows the relation of the horizontal location of the condylar head in the sagittal plane, showed almost no change in both groups, and no significant difference was shown in this study [2]. This showed that anterior and posterior locations of the condylar head had almost no change at T1. S–N, which shows the relation of the vertical location of the condylar head, showed a significant difference at T1 in both groups. It was decreased at T1 and increased at T2, which showed a tendency to return to the original value preoperatively. There was no significant difference at T0 and T2, and this showed that the condylar head moved backward at T1 but returned to its original location at T2. S–M and S–N values measured in the sagittal plane showed no statistically significant difference in both groups at any of the three time points, and the pattern of change was identical in both groups.

Choi et al. [24] reported that condylar angle in the coronal plane was reduced to 65% on the right side and 50% on the left side [24]. In addition, Kim et al. [23] reported that the condylar head angle in the coronal plane was reduced by an average of 0.92° identically on both sides. Moreover, in this study, the condylar head angle in the coronal plane (B-angle) decreased at T1 in both groups, which corresponded to the results of previous studies. The B-angle showed rotation of the condylar head in the coronal plane, which showed that the condylar head rotated outwardly. Because the segment has maximum contact when conducting rigid fixation after separating the bone into proximal and distal segments during orthognathic surgery, the proximal segment rotates outward in the coronal plane. The values of the two groups increased at T2, and the pattern returned to its original state.

Lee et al. [25] reported that there was a clearly significant difference between the distances of condylar heads preoperatively and postoperatively in the nonsevere versus severe asymmetry groups. Moreover, the significant difference in horizontal distance of the condylar head was clearer in the nonsevere versus the severe asymmetry groups.

In this study, the distance between both condylar heads in the coronal plane (C-distance) increased in both groups at T1, but a significant difference was shown in group B with a small MD. This is because the distal segment does not rotate horizontally when the mandible setbacks in group B, where MD is not severe, and the proximal segment rotates inward to the space between the proximal and distal segments on both sides and the mandibular flaring increases the distance between the condylar heads.

On the other hand, in group A with severe asymmetry, the condylar heads can be returned more manually to their original location by posterior bending osteotomy (PBO), so that less inward rotation of the proximal segment occurs. A significant difference was shown in group B at T2 and decreased again to return to the preoperative location.

In this study, there was almost no difference in the amount or pattern of condylar head change between groups with large and small MD, and the distance between condylar heads in the coronal plane showed a more stable change in pattern in the group with large MD. Therefore, it can be said that accurate measurement and analysis preoperatively is required to reduce change in the proximal segment during orthognathic surgery regardless of the degree of asymmetry. Furthermore, effort should be put on the selection of condylar head location when fixing proximal and distal segments to minimize relapse due to condylar head displacement postoperatively. PBO was conducted on patients with the possibility of a large change in condylar head location, and removal of contact interruption through additional procedures had a positive influence on conserving condylar head location.

In this study, CBCT was used in restricted patients at T0, T1, and T2 to observe the pattern of condylar head location change. Tracing research with longer periods is required because there is a possibility that condylar head location can change in 6–12 months postoperatively. The amount of skeletal movement in the surgical plane, preoperative direction of the condylar head, and choice of surgical method should be observed, and changes in these values should be investigated in more number of patients than included in our study to obtain more generalized results.

## Conclusions

This study was conducted on 30 patients hospitalized for orthognathic surgery because of prognathism or facial asymmetry. Condylar head location and pattern were analyzed by CBCT at T0, T1, and T2. Through the review of change in condylar head location, we concluded that (1) in the axial plane, the condylar head rotated inward at T1 in both groups but tended to return to its original locations at T2. (2) In the sagittal plane, there was almost no anterior–posterior change at T0 and T2 in both groups. However, it moved vertically downward at T1 and tended to return to its original location at T2. (3) In the coronal plane, condylar head rotated outward at T1 in both groups but tended to return to its original location at T2. Distance between the condylar heads in the coronal plane increased in both groups, and a significant difference was shown in the group with a small MD.

In summary, there was almost no difference in the amount or pattern of condylar head location change between the groups with large and small MD after orthognathic surgery.
